# Determination of essential phenotypic elements of clusters in high-dimensional entities—DEPECHE

**DOI:** 10.1371/journal.pone.0203247

**Published:** 2019-03-07

**Authors:** Axel Theorell, Yenan Troi Bryceson, Jakob Theorell

**Affiliations:** 1 IBG-1: Biotechnology, Institute of Bio- and Geosciences, Forschungszentrum Jülich GmbH, Jülich, North Rhine-Westphalia, Germany; 2 Center for Hematology and Regenerative Medicine, Department of Medicine Huddinge, Karolinska Institutet, Stockholm, Sweden; 3 Broegelmann Research Laboratory, Department of Clinical Medicine, University of Bergen, Bergen, Norway; 4 Nuffield Department of Clinical Neurosciences, University of Oxford, Oxford, United Kingdom; New Jersey Institute of Technology, UNITED STATES

## Abstract

Technological advances have facilitated an exponential increase in the amount of information that can be derived from single cells, necessitating new computational tools that can make such highly complex data interpretable. Here, we introduce DEPECHE, a rapid, parameter free, sparse k-means-based algorithm for clustering of multi- and megavariate single-cell data. In a number of computational benchmarks aimed at evaluating the capacity to form biologically relevant clusters, including flow/mass-cytometry and single cell RNA sequencing data sets with manually curated gold standard solutions, DEPECHE clusters as well or better than the currently available best performing clustering algorithms. However, the main advantage of DEPECHE, compared to the state-of-the-art, is its unique ability to enhance interpretability of the formed clusters, in that it only retains variables relevant for cluster separation, thereby facilitating computational efficient analyses as well as understanding of complex datasets. DEPECHE is implemented in the open source R package DepecheR currently available at github.com/Theorell/DepecheR.

## Introduction

Since the introduction of the first single colour flow cytometers in the 1960s, there has been a remarkable increase in the complexity of data that can be generated with single-cell resolution. Currently, flow and mass cytometers able to simultaneously assess up to 40 cellular traits are becoming widely available [1]. In parallel, the development of high-throughput sequencing technology has facilitated single-cell transcriptomic analyses gauging expression of thousands of distinct transcripts [2]. Furthermore, development of high-resolution single-cell proteomic analyses are underway, similarly providing highly complex datasets [3].

These technological advances necessitate new computational approaches to analyses of multi- and megavariate single cell data [4–7]. Previous algorithms have contributed to automating analyses, thereby enhancing reproducibility and avoiding a need for *a priori* biological knowledge. Thus, these algorithms have major advantages over the classical analysis strategies of cytometry data that are based on manually defined uni- or bivariate filters, commonly referred to as gates [8]. Automated analysis algorithms, not restricted to uni- or bivariate displays of the data, have also made it possible to extract much more of the information embedded in multivariate data. Furthermore, as they generally scale well, automated analysis algorithms can also be applied to datasets where manual gating is impossible, such as single-cell transcriptomic data with thousands of transcripts assessed for each cell. To date, however, analysis strategies based on manual gating still dominate where they can be used and are still considered as the gold standard for cytometry data analysis. One likely reason for this is that cell types defined by manual gates are easy to comprehend, as they are defined by few markers. In an attempt to combine the objectiveness, reproducibility and scalability of automated analysis pipelines with the high interpretability of manual gating strategies, we have developed an algorithm termed Determination of Essential Phenotypic Elements of Clusters in High-dimensional Entities (DEPECHE). DEPECHE simultaneously clusters and simplifies the data by identifying the variables that contribute to separate individual clusters from the rest of the data. We have implemented DEPECHE in R [9] (in the open source package DepecheR), providing a complete software suite for statistical analysis and visualization of single cell omics data.

## Results and discussion

At its core, DEPECHE uses a penalized k-means clustering algorithm, related to the standard k-means algorithm [10]. Both k-means and penalized k-means clusters data by fitting *k* cluster centers to the data. In k-means, the fitting objective is to minimize the sum of squared distances from each data point to its closest cluster center. In penalized k-means, the k-means objective has been complemented by a penalization objective which minimizes the sum of the L1-norms of the cluster centers, thereby moving them towards the origin.

**Fig 1:**
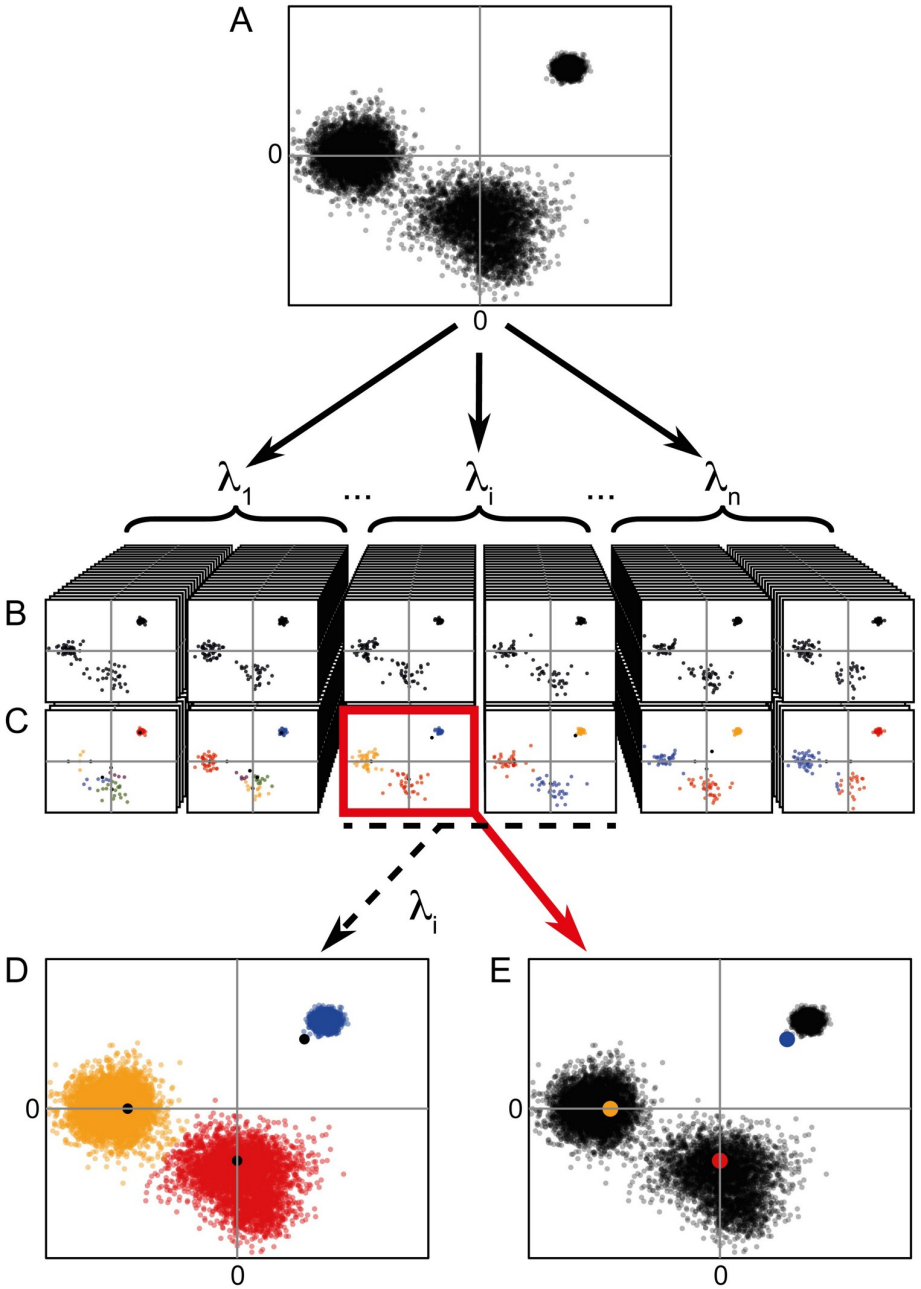
Illustration of the DEPECHE workflow. A) The original dataset *D*. B) Resampled datasets with *N*_*r*_ data points per dataset, are generated by sampling data points from *D* with resampling. Each resampled dataset has a corresponding penalty *λ*_i_ (*i = 1*…*N*_*λ*_). C) Each dataset in b is clustered with sparse k-means, using its corresponding penalty *λ*_i_. The red frame highlights the clustering with the strongest attractors, *i*.*e*. the most generalizable solution (see Methods, “Simultaneous Clustering and Parameter Tuning”). D, E) Finally, the full dataset is clustered. In cases where it is computationally feasible, the full data set is re-clustered with the optimal penalty (D). Otherwise, the final clustering is produced by allocating each data point to its closest cluster center, using the most generalizable cluster center solution produced with the optimal penalty in B (E).

Generally, when measuring a large number of biological variables, observations tend to agglomerate around certain positions in some variables, whereas in other variables, the observations are spread more or less evenly. Due to their even spread, the latter variables can be considered uninformative. In this situation, the dual clustering objective of penalized k-means will fit the cluster centers to the observation agglomerates in the informative dimensions, whereas the penalty will draw the cluster centers to the origin in the uninformative dimensions and thus discard them. The resulting cluster definitions are referred to as sparse, as the clusters are only defined in the informative dimensions. The relative importance of the k-means objective and the penalization objectives in the penalized k-means algorithm is controlled by the penalty parameter *λ*. A high penalty *λ* implies that many variables will be considered uninformative, implying that many variables will be discarded. When all variables of a cluster are discarded, the cluster is discarded too. Thus, a high penalty *λ* results in high sparsity and a low cluster resolution, and vice versa [11] (see Methods, “Clustering with DEPECHE”). A key feature of penalized k-means is that the penalty addresses each variable in each cluster center independently, implying that different sparsity patterns will emerge from distinct clusters. This feature differentiates penalized k-means from sparse k-means [12] and regularized k-means [13], which only identify variables that are uninformative for all clusters. To illustrate this, a 10,000 point, 11-variate dataset with 10 normally distributed clusters was constructed. In this dataset, 10 of the variables contributed to the separation of two clusters (S1 Fig). In this example, sparse k-means could only exclude the single variable, which did not define any clusters, whereas DEPECHE correctly excluded all nine dispensable variables from each cluster individually. Note that if *k* is chosen so large that at least one cluster is discarded, the resolution of the emerging clusters depends entirely on the magnitude of the penalty *λ* and not on *k*, since DEPECHE redistributes observations belonging to clusters that are pulled to the origin to the remaining clusters. In DEPECHE, the penalty *λ* is tuned to identify the most *reproducible* clustering resolution, here termed the “optimal resolution” [13]. To illustrate what we mean by *reproducible*, we constructed an example, featuring a bi-variate dataset *D* (Fig 1A). Visually, dataset *D* contains three clusters, where the centers of the two larger clusters are located close to either axis. For these two clusters, one variable is sufficient to define their position. Now, if multiple datasets were generated from the same data source as *D*, for example by repeated experiments, we assume that they would contain the same clusters. Hence, the optimal penalty *λ*_i_ (that corresponds to the optimal resolution of *D*) is defined as the penalty for which the resulting clusters have the highest similarity, where similarity is measured in the Adjusted Rand Index (ARI) [14] (see Methods, “Tuning the penalty”), when clustering the generated datasets independently. In contrast, when clustering the same datasets with a penalty *λ* that differs significantly from the optimal penalty, the stochastic differences between the datasets are likely to induce solutions that deviate in cluster number, number of defining variables, and cluster center positions. In practice, DEPECHE tests a range of penalty values, (*λ*_1_*<⋯<λ*_N*λ*_), each on a collection of dataset pairs which are generated by sampling *N*_r_ data points from *D* (Fig 1B) with resampling. The optimal resolution is defined as the penalty *λ*_i_, which yields the lowest average variability within each dataset pair, as measured by the ARI. In our example, this corresponds to the penalty *λ*_i_ that yields 3 clusters, since 3 similar clusters are identified in each resampled dataset of *D* (Fig 1C). The penalties *λ*_1_ and *λ*_N*λ*_ are considered suboptimal, since with these penalties, the stochastic differences in the resampled datasets lead to less coherent clustering results compared to results obtained with the optimal penalty *λ*_i_. From here (Fig 1C) DEPECHE uses two alternative routes. If the dataset *D* has few data points, the full dataset *D* is clustered using the optimal penalty *λ*_i_ (Fig 1D). If instead the number of data points in the dataset *D* is high (default in DepecheR >10^4^), the most generalizable cluster centers that were produced using the optimal penalty are chosen (see Methods, “Simultaneous Clustering and Parameter Tuning”) and the data points of *D* are allocated directly to their closest cluster center (Fig 1E). This default setting enables rapid iterated clustering of large datasets possible (see calculation times in S2 Fig).

**Fig 2:**
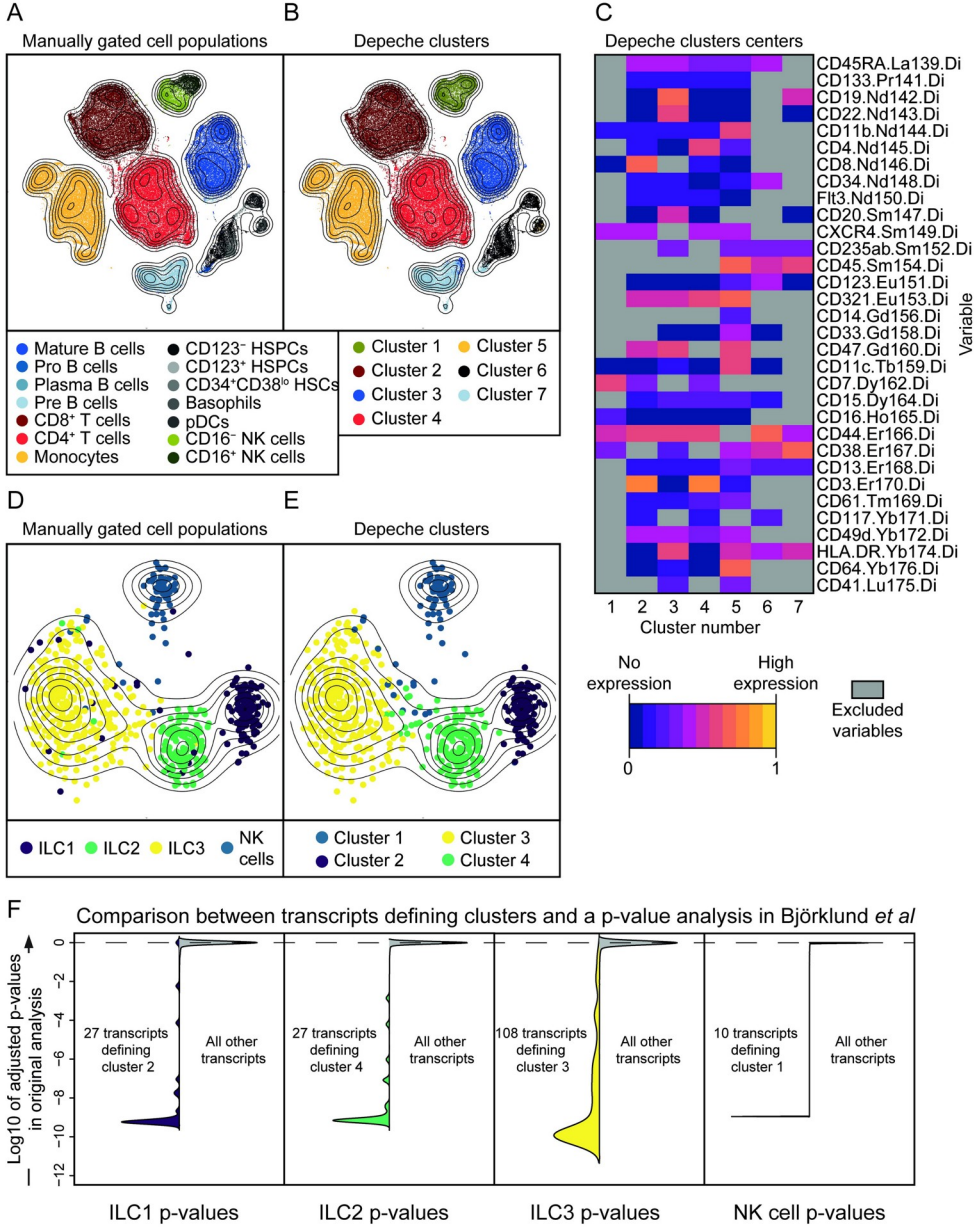
DEPECHE performance with real datasets with 32 or 35177 variables. A-B) bi-variate t-distributed stochastic neighbor embedding (tSNE) representation of the 32-variate mass cytometry data. A) distribution of manually defined cell populations over the tSNE field. B) distribution of DEPECHE clusters over the tSNE field. C) Heatmap of the expression pattern at each cluster center. Light colors show that the marker is highly expressed at the cluster center, whereas dark colors shows the opposite. Grey color indicates that the variable in question does not contribute to defining the cluster. For Fig a-c all, 104184 cells have been clustered. D-E) tSNE representation of the 137-variate data subset that could efficiently distinguish the clusters in the 35177-variate single-cell transcriptome dataset. D) distribution of the cell types defined by index-sorting and manual gating on protein expression profiles shown over the tSNE field. E) distribution of DEPECHE clusters over the tSNE field. F) Violin plots illustrating the overlap between the original analysis by Björklund *et al* and the DEPECHE analysis. For each subplot, the left and right side illustrate the distribution of the transcripts defining the clusters, and all other transcripts, respectively. The y-axis shows the log10 of the p-values in the original analysis adjusted for multiple comparisons. For Fig D-F, all 648 cells have been clustered.

To evaluate how biologically accurate DEPECHE clustering is on mass cytometry data, a 32-variate mass cytometry bone marrow dataset [14] was clustered, and the overlap to 14 visually pre-defined cell populations was quantified using the ARI. With this dataset, DEPECHE identified 7 clusters at the optimal resolution, corresponding to all large pre-defined cell populations and to agglomerates of smaller cell populations, rendering an average ARI of 0.96, where an ARI of 1 corresponds to exact reproduction and an ARI of 0 means that the produced clusters are no more accurate than random allocation (Fig 2A and 2B, S2A Fig). Furthermore, using the DEPECHE algorithm, the number of variables defining each cluster was reduced from 32 to a range from 8 to 28, thereby enhancing interpretability (Fig 2C). When comparing to other state-of-the-art clustering algorithms [14–19], DEPECHE obtained similar ARI as the best algorithms for both the 32-variate dataset and another 14-variate, 24 population, mass cytometry dataset [20] (S3A Fig, Table 1).

**Table 1:** Background information on all datasets.

Dataset	Data origin	n cells	n variables in analysis	n clusters in original
Levine[14]	Mass cytometry	104184	32	14
Bendall[20]	Mass cytometry	81747	14	24
Björklund[21]	scRNAseq	648	35177	4
Biase[5]	scRNAseq	56	19571	3
Deng[5]	scRNAseq	268	13867	10
Goolam[5]	scRNAseq	124	15487	5
Kolodziejczyk[5]	scRNAseq	704	15117	3
Pollen[5]	scRNAseq	301	13860	11
Yan[5]	scRNAseq	90	13608	7

**Fig 3:**
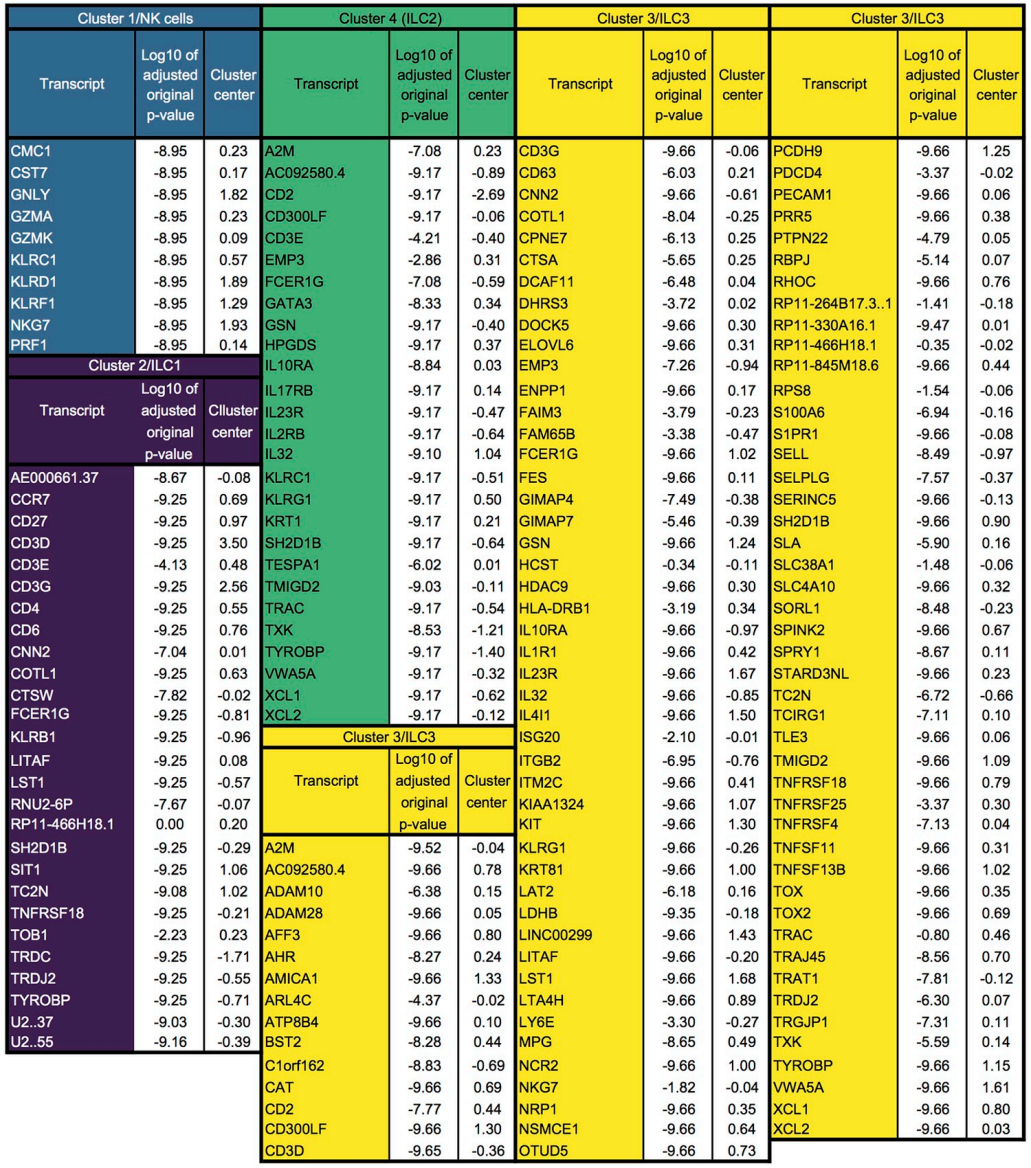
Transcripts defining clusters in Björklund *et al* dataset. Blue background color indicates 10 transcripts associated with cluster 1/NK cells. Violet background color indicates 27 transcripts associated with cluster 2/ILC1. Green background color indicates 27 transcripts associated with cluster 4/ILC2. Yellow color indicates 108 transcripts associated with cluster 3/ILC3.

Single-cell transcriptomic datasets feature tens of thousands of variables. Thus, compared to cytometry datasets, the need to exclude variables of low interest is even more pressing. We therefore evaluated DEPECHE’s ability to cluster and extract the key transcripts defining clusters of a previously published single-cell transcriptomic dataset (Fig 2D–2F) [21]. In this dataset, a total of 648 ILC1, ILC2, ILC3 and NK cells from three donors’ tonsils were index-sorted prior to RNA sequencing. Hence, these cell types, defined by expression of specific sets of markers using manual gating, can be compared to clusters unbiasedly determined by RNA expression profiles [21]. In the DEPECHE analysis, no pre-selection of transcripts was performed, and hence, 35177 unique transcripts were included for each of the 648 cells. With the optimal penalty *λ*, four clusters were identified (Fig 2E). These corresponded well to the cell types as defined by protein expression; 84, 97, 91 and 97 percent of ILC1, ILC2, ILC3 and NK cells sorted into separate clusters, respectively (Fig 2D and 2E, S2B Fig), leading to an average ARI of 0.78, which was comparable to clusters obtained in the original publication [21]. Notably, cluster 1–4, corresponding to NK cells, ILC1, ILC3 and ILC2, were defined by 10, 27, 108 and 27 transcripts, respectively (Figs 2F and 3), leading to a 99.9% average decrease in the number of variables. The transcripts identified to define the clusters in our analysis corresponded to those identified as most differentially expressed in the original study [21] (Fig 2F). Thus, by identifying a finite number of variables, DEPECHE analysis can increase interpretability and aide down-stream analyses. When DEPECHE clustering was compared to that of state-of-the-art algorithms [5–7] on the aforementioned dataset as well as six other datasets (see Table 1), it consistently performed well as indicated by ARI (S3B Fig). Thus, when applied to megavariate single-cell transcriptomic data, DEPECHE identifies clusters corresponding to known cell types and reduces the complexity of the results thousand-fold by finding a subset of markers that define each cluster in a way that compares well to previous knowledge. Notably, as DEPECHE selects the most robust identifiers of each cluster, some potentially interesting transcripts might be excluded by DEPECHE clustering, *e*.*g*. transcripts that are highly expressed in a minority, but lacking from a majority of cells in a cluster.

In conclusion, DEPECHE turns the penalized k-means methodology into a parameter free analysis technique guided by efficient calculation of the optimal clustering resolution. By doing so, it can simultaneously address the problems of finding biologically relevant clusters and identifying specific variables that define these clusters. This is crucial in order to comprehend the noisy and often over-complicated data generated with current single cell technologies.

## Methods

### Clustering with DEPECHE

Clustering in DEPECHE is performed using a penalized version of the k-means algorithm, which is related to the k-means algorithm [10]. In this section, the k-means algorithm is outlined, followed by an explanation of how it is extended to penalized k-means.

The k-means algorithm clusters data by fitting a mixture of normal distributions to the data with *k* equal mixture components and unit variance. Formally, *k d*-dimensional cluster centers, denoted μ_m,j_ where *m = 1…k* and *j = 1…d*, are fitted to the *n d*-dimensional datapoints *x*_l,j_, where *l = 1…n*, by maximizing the score function

$$Q=\sum_{m=1}^{k}\sum_{l=1}^{n}z_{i,l}\sum_{j=1}^{d}{\left(x_{l,j}-\mu_{m,j}\right)}^{2},$$ (1)

where *z*_m,l_ is 1 if the *l*^th^ data point belongs to the *i*^th^ cluster and zero otherwise. The score *Q* is optimized using an Expectation Maximization (EM) algorithm [22], *i*.*e*. so called E- and M-steps are iterated alternatingly until the score *Q* stops improving. In the E-step, the allocation variables *z*_m,l_ are updated so that each data point is allocated to its closest cluster according to the Euclidean norm. That means that *z*_m,l_ = 1 if the *m*^th^ cluster center is the cluster center closest to the *l*^th^ data point and *z*_m,l_ = 0 otherwise [23]. In the M-step, each cluster center μ_m,j_ is moved to the center of the data points allocated to it. When no more reallocation occurs in the E-step, the algorithm has converged.

In order to reduce the influence of uninformative dimensions that only contribute with noise, penalized k-means introduces an L1-penalty for each element of each cluster center μ_m,j_ to the optimization objective. Formally, the score function *Q* in Eq (1), is updated:

$$Q=\sum_{m=1}^{k}\sum_{l=1}^{n}z_{m,l}\sum_{j=1}^{d}{\left(x_{l,j}-\mu_{m,j}\right)}^{2}-\lambda \sum_{m=1}^{k}\sum_{j=1}^{d}\left|\mu_{m,j}\right|,$$ (2)

where *λ* is a positive penalty term. The additional term in the score function, introduced in Eq (2) results in a change in the M-step of the original EM-algorithm of the k-means algorithm. Keeping *z*_m,l_ for all *l* fixed and optimizing *Q* with respect to μ_m,j_, the M-step is:

$$\mu_{m,j}=sign(\frac{\sum_{l=1}^{n}z_{m,l}x_{l,j}}{\sum_{l=1}^{n}z_{m,l}})\cdot \text{max}\left(|\frac{\sum_{l=1}^{n}z_{m,l}x_{l,j}}{\sum_{l=1}^{n}z_{m,l}}|-\frac{\lambda }{2\sum_{l=1}^{n}z_{m,l}},0\right)$$ (3)

Depending on the choice of the penalty parameter *λ*, some components of some clusters centers will be set to 0 in the M-step. Note that penalized k-means with penalty *λ* = 0 reduces to the original k-means algorithm.

In DEPECHE, cluster centers that are moved to the origin in the M-step are eliminated and not assigned any data points in the E-step. Due to the elimination of clusters, the number of produced clusters is independent of *k* and dependent on the penalty *λ* as long as at least one cluster is eliminated. In DEPECHE, *k* is always chosen to be so large that at least one cluster is eliminated. Eq (2) is a special case of the penalized model based clustering algorithm by Pan and Shen with unit variance and equal mixture components [11]. By imposing the penalty for each dimension and each cluster, penalized k-means identifies the dimensions that do not distinguish a particular cluster from the rest of the data, thus leaving these dimensions out of the definition of that cluster.

Penalized k-means, as well as k-means, relies on a procedure for initializing the positions of the cluster centers. Therefore, in DepecheR, the initial cluster positions are generated using the well performing seed generation algorithm of k-means++ by Arthur and Vassilvitskii [24]. The early iterations of the EM-algorithm are particularly delicate in DEPECHE, due to the elimination of clusters at the origin in the E-step. Poor initialization of the clusters, in combination with a high penalty *λ*_i_, might lead to elimination of too many clusters in the early E-steps, yielding fewer clusters in the end result than necessary to optimize *Q*. To remedy this, DEPECHE starts each run of penalized k-means, regardless of the chosen *λ*_i_, with penalty *λ* = 0. The penalty is then increased linearly over a number of E-steps until it reaches the predetermined value *λ*_i_.

The EM-algorithm guarantees convergence to an optimum of the score *Q*, but not necessarily to the global optimum. In order to diminish the influence of the starting state, the EM-algorithm is run several times with random initialization, and the solution with optimal score *Q* is chosen. For penalty optimization, the number of runs is determined according to a set of rules that are outlined in the section “Tuning the penalty”. For producing the final results (Fig 1E), a fixed number of runs is performed (21 (3 x (8–1)) processor cores) runs is the default in DepecheR). To further decrease stochastic variability in the computational results, *k* is set considerably higher than the expected number of final clusters, which also diminishes the sensitivity to the starting state. In the extreme case where *k* is set equal to the number of data points *n*, the outcome of penalized k-means is deterministic.

### Tuning the penalty

In this section, we describe the optimization scheme which is used for tuning the linear penalty *λ*. The outline of the algorithm:

1. Choose a wide and exponentially distributed range of penalty terms *λ*_i_, *i = 1*..*N*_*λ*_, that are considered for clustering the dataset *D*.
2. Create 2 datasets per penalty term *λ*_i_, called *D*_1,i_ and *D*_2,i_, by sampling *N*_r_ data points from *D* with replacement.
3. Run the penalized k-means algorithm on the datasets *D*_1,i_ and *D*_2,i_, yielding sets of cluster centers, denoted *Μ*_1,i_ and *Μ*_2,i_.
4. Create the partitions *P*_1,i_ and *P*_2,i_, by allocating all data points of the dataset *D* to their nearest cluster center of the sets *Μ*_1,i_ and *Μ*_2,i_. The allocation is equivalent to one E-step of the k-means algorithm.
5. Determine the Adjusted Rand Index (ARI), denoted *r*(*λ*_i_) from *P*_1,i_ to *P*_2,i_ [25].
6. Repeat step 2–5 and average the obtained ARIs *r*(*λ*_i_) penalty wise until a stopping criteria regarding the statistical certainty of the obtained ARIs *r*(*λ*_i_) is met (remark 2, section “Tuning the penalty).
7. Choose the optimal penalty *λ*_i_, which is the penalty with the largest ARI *r*(*λ*_i_).

Some remarks:

1. To increase the likelihood of finding he optimal penalty value in the pre-defined range, datasets with very high kurtosis are log2-transformed and all datasets are divided by their total standard deviation prior to sampling. With this setup, all tested datasets have their optimal penalty in the default range. However, new datasets might have other requirements for penalties. In cases where the most extreme low or high penalty value is selected by the optimization procedure, DepecheR will warn the user, and suggest another range of penalties, or a larger sample size. The default penalty range in DepecheR is 2^0^, 2^0,5^… 2^5^.
2. The repetition of Step 6 is necessary, since the obtained ARI *r*(*λ*_i_) is a random variable, due to the random procedure for creating the datasets *D*_1,i_ and *D*_2,i_ and the random procedure for initializing the penalized k-means algorithm. To determine the necessary number of runs, after an initial fixed number of runs (default 20 in DepecheR), DEPECHE uses three stopping criteria: The first criterion creates an interval of width 2 standard errors around the obtained mean of *r*(*λ*_i_) and checks if the interval around the optimal ARI *r*(*λ*_i_) has a zero overlap with the other intervals. The second criterion checks whether the standard error of the mean of *r*(*λ*_i_) for the optimal penalty *λ*_i_ is below a threshold. The third criterion terminates the process after a maximal number of runs (default 100 in DepecheR).
3. Step 2 requires a samples size *N*_r_. A natural choice is to set *N*_r_ equal to the number of data points, n. However, in cases where n is very large, so that the computational load of the optimization scheme becomes limiting, it is preferable to choose a smaller *N*_r_. In DepecheR, *N*_r_ = 10^4^ by default, in case *n*≥10^4^. Notice that when an optimal penalty *λ*_i_ is discovered using sample size *N*_r_≠*n*, the corresponding optimal penalty when sampling the full dataset *D* with magnitude *n* is (approximately) *λ*_i_ ⋅ *n*/*N*_r_, since the attraction force of a cluster is proportional to the number of data points in it.
4. In step 5, the ARI is chosen as similarity measure between the partitions, since it corrects for chance. This means that it gives a zero similarity for trivial partitions, such as having all data points in a single cluster. Exact calculation of the ARI is computationally intractable for large datasets. Therefore, DEPECHE relies on an approximate ARI computation, based on 10^4^ random pairs of data points.

### Simultaneous clustering and parameter tuning

For very large datasets (*n*>10^8^), not only the penalty optimization, but also the final clustering once the optimal penalty has been found, may be computationally intractable. However, increasing the size of the dataset does not necessarily lead to an increase in number of clusters at the optimal resolution. In this case, it is feasible to cluster a subset of the full dataset *D* to obtain cluster centers *M* and then allocate the remaining data points of *D* to their closest clusters in *M*. This boosts computational efficiency since allocation imposes a much smaller computational load than clustering. Since several subsets of *D* are produced and clustered during the tuning of the penalty parameter **λ**, it is computationally favorable to retrieve cluster centers *M* that were produced during the parameter tuning and use them to cluster *D*.

When picking a set of cluster centers *M* from the penalty tuning, the question arises which set of centers *M* to take, since several sets of centers, denoted *M*_i,p_ (*p = 1*…*N*_p_), are produced for the optimal penalty *λ*_i_. In DEPECHE, the centers *M*_i,p_ that have the strongest similarity (on average) to the remaining *N*_p_-1 centers is chosen and is referred to as the most generalizable cluster set. The level of similarity between the centers *M*_i,p_' and *M*_i,p_'' is quantified using the ARI between the partitions *P*_i,p_' and *P*_i,p_'', induced by allocating each data point of *D* to its closest cluster center in *M*_i,p_' and *M*_i,p_'' respectively.

#### Empiric performance of the penalty tuning scheme

Roughly speaking, DEPECHE combines a flavored penalized k-means algorithm with a parameter tuning scheme, which identifies an optimal resolution. A naturally arising question is then whether the parameter tuning scheme is able to determine a biologically relevant resolution or if other penalized k-means clustering resolutions outperform the resolution chosen by DEPECHE. Using a range of datasets (Table 2), the biological relevance (measured in ARI to the manually curated solution) of the optimized DEPECHE partitions were compared to the biologically optimal partition among all partitions generated with 20 repetitions on each of a range of 11 penalties per dataset. Overall, the DEPECHE resolution-selection showed close to optimal performance, as the selected solutions only had a median of 0.02 lower ARI to the gold standard (range 0–0.065) than the best possible solution with all penalties (Table 2).

**Table 2:** ARI between DEPECHE partitions and golden standard partitions.

Dataset	Median ARI in S2 Fig	Maximal ARI with any penalty	Difference
Levine[14]	0.961	0.975	0.015
Bendall[20]	0.841	0.873	0.032
Björklund[21]	1	1	0
Biase[5]	0.782	0.842	0.06
Deng[5]	0.827	0.848	0.021
Goolam[5]	0.629	0.639	0.009
Kolodziejczyk[5]	0.992	1	0.008
Pollen[5]	0.863	0.928	0.065
Yan[5]	0.626	0.691	0.064

### Transforming and centering the data

The clusters produced by DEPECHE, as well as their interpretation, depends on the transformation and centering of the data. The transformation determines the relative importance of the measured variables, where variables with a larger spread have stronger influence on the clustering. The centering defines where zero occurs in each variable, thereby influencing the clustering results due to the linear penalty.

DEPECHE is applicable to a large range of datasets where the numbers of dimensions, *d*, and the number of data points, *n*, can vary with many orders of magnitude. The differing characteristics of these datasets require different treatments with respect to transformation and centering.

#### Transformation

Empirically, a majority of single-cell transcriptome datasets tend to have a few variables where the variance is many orders of magnitude greater than in the other variables. In this case, the high-variance variables will *de-facto* determine the clustering, implying that the clustering will fail to take the majority of the measured information into account. To even out the influence of these high-variance variables on the clustering outcome, the data is log transformed when such variables are present. In DepecheR, this data behavior is detected automatically by concatenating all variables into a one-dimensional vector, for which the kurtosis is calculated. A high kurtosis indicates that the variables differ greatly in their variance. For datasets with low kurtosis, refraining from the log transform is preferable to avoid unnecessary data distortion.

#### Centering

Centering the origin to be close to the bulk of the data is preferable, in order to have all biological clusters at approximately the same distance from the origin. Having some biological clusters close to the origin and some far off is often unwanted, since the linear penalty then imposes a preference for creating clusters close to the origin. Apart from influencing the clustering, the centering also determines the interpretation of the obtained sparsity. Just as for scaling, which centering scheme to apply depends on the dataset.

For low dimensional datasets (*d*>100), DEPECHE applies maximal density centering, which sets the zero in each dimension to coincide with the highest data density. The density it computed by collecting the data in equally spaced bins (default number of bins in DepecheR is the number of data points *n* divided by 50), where the bin with the highest number of data points has the highest density. Using this scheme, sparsity (*i*.*e*. that a variable is non-contributing to the definition of a cluster) is interpreted as non-deviance from the most common outcome. It also ensures that the origin is relatively close to the bulk of the data, since it is located at the most common outcome for each variable respectively. The benefit of this scheme is that it boosts sparsity, by declaring the most common outcome non-defining. However, for high dimensional datasets (*d*≥100), maximal density centering can push the origin so far away from the center of mass of the dataset, that the penalty starts to impose an unwanted, artificial influence on the clustering, hampering the biological relevance of the clusters. To avoid this, DEPECHE imposes a mean centering scheme for such datasets, which locates the origin at the center of mass of the dataset.

A potential complication, related to centering, occurs when a biologically relevant cluster is located very close to the origin, since DEPECHE creates no clusters in the origin and will then force the cluster to merge with other clusters. However, this scenario was never detected in real data.

### Experimental procedures

#### Generation and analysis of synthetic example for k-means algorithm comparisons

The synthetic dataset was generated using base package functions. The standard deviation was identical for all clusters and dimensions. For classical k-means analysis, the k-means function in the stats package was used. For sparse k-means, the sparcl package was used [26].

#### Preprocessing of mass cytometry data

The benchmark datasets from Levine *et al* [14] and Bendall *et al* [20] were transformed using the flowTrans package [27] before used in any clustering algorithm.

#### Preprocessing of single-cell transcriptomic data

The dataset from Björklund *et al* [21] was normalized using the sva package [28] as in the original manuscript. For this dataset, non-expressed transcripts were removed, lowering the number of variables from 64443 to 35177.

The gold-standard datasets used for benchmarking in the publication by Kiselev *et al* [5] were obtained in a pre-processed state. Before clustering with any algorithm, the gene filter function used in the sc3 package was used [5], with settings removing the genes that were expressed in more than 90% of the cells. This resulted in the number of transcripts presented in Table 1 (range 13608–19571 transcripts).

### Code availability

All code necessary to generate the figures and tables in the manuscript are included in S1 File. The software package DepecheR is available for download at (https://github.com/Theorell/DepecheR).

## Supporting information

S1 Fig A comparison of classical K-means, sparse K-means and DEPECHE. A: Overlap between true clusters and clusters generated with classical k-means, sparse K-means, or DEPECHE. Numbers in heatmaps denote percent of the true cluster present in the generated cluster in question. Red color indicates high overlap, blue color low overlap. B: Total loadings for all input vectors with sparse k-means. Light color indicates a strong contribution to separation of the clusters and vice versa. Grey color indicates that the variable has been excluded. C: Cluster center matrix for DEPECHE analysis. Rows indicate the DEPECHE clusters, columns indicate the variables that contribute to separating at least one cluster. A light color indicates that the cluster center is located in the upper part of the distribution of values in the vector in question, and vice versa. Grey color indicates that the variable has been excluded. For DEPECHE, only 10 dimensions are shown, as the 11^th^ did not contribute to separating any cluster.
(TIF)

S2 Fig Heatmaps comparing the golden standard partitions to the DEPECHE partitions for A) the 32-variate Levine dataset and B) the 35166-variate Björklund dataset. Red color indicates large overlap, blue color indicates low overlap between a gold standard-vs-DEPECHE cluster pair. Numbers in heatmaps denote percent of the golden standard cluster present in the DEPECHE cluster in question.
(TIF)

S3 Fig Algorithm comparisons. For all graphs, the x-axis shows the algorithms and the y-axis shows the Adjusted Rand Index comparing the clustering result with the golden standard clustering. Below each graph is the average computational time in seconds for the benchmarking performed on a laptop computer with 4 2.8 GHz Intel Core i7 processors. A) Subsamples with 20000 unique cells from two mass cytometry datasets published by Levine *et al* and Bendall *et al* were clustered with DEPECHE and six previously published algorithms. For each dataset and algorithm, clustering was performed on 20 unique subsamples. For flowClust, flowPeaks and SamSPECTRAL, that do not perform internal parameter tuning, a range of parameter values were evaluated and the parameter value sets generating the highest ARI values were selected for display. B) The full Björklund dataset, as well as six other datasets previously used for benchmarking by Kiselev *et al* were clustered 20 times with DEPECHE and three other algorithms. The Björklund dataset was normalized to reduce batch effects, with the procedure described in the original publication. These six datasets were also automatically log2-transformed within DEPECHE, and thus, log2-transformation was applied also for Sincera and pcaReduce, whereas sc3 was fed both log2- and untransformed data. The lower and upper hinges of all boxplots extend to the 25:th and 75:th percentile, whereas the line in the middle describes the median. The whiskers extend to the lowest and highest value no further than 1.5 times the distance between the 25:th and 75:th percentile. Outside of this range, the observations are considered outliers and are shown as dots.
(TIF)

S1 File The code used generate all figures. (ZIP)

S2 File Information on how to retrieve the data used for this study. (PDF)
