# Supplementary figures and images for: Determination of essential phenotypic elements of clusters in high-dimensional entities—DEPECHE
Source: PLoS One. 2019 Mar 7;14(3):e0203247. doi: 10.1371/journal.pone.0203247 (PMC6405191; doi:10.1371/journal.pone.0203247)

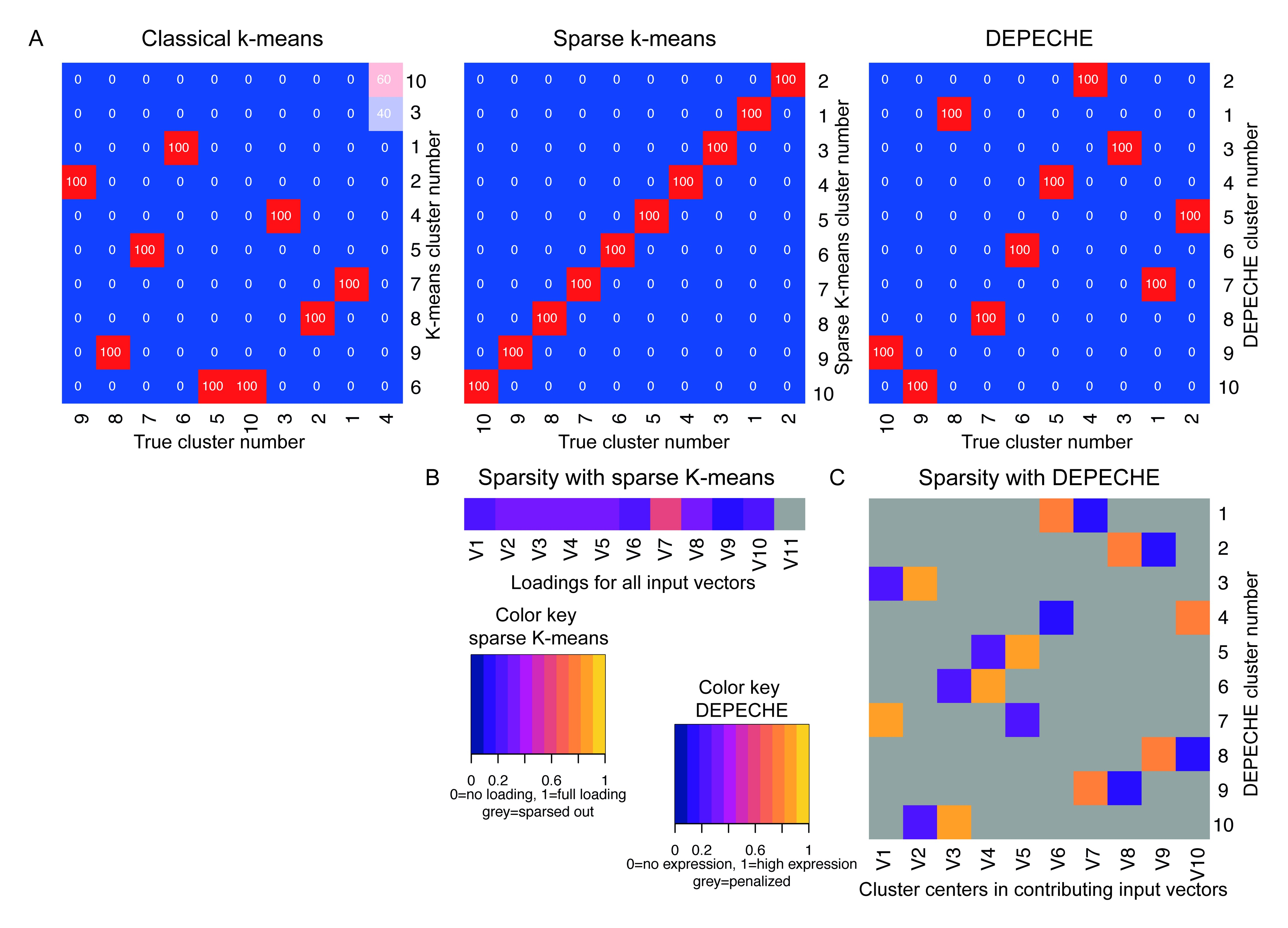

Supplement: S1 Fig — A: Overlap between true clusters and clusters generated with classical k-means, sparse K-means, or DEPECHE. Numbers in heatmaps denote percent of the true cluster present in the generated cluster in question. Red color indicates high overlap, blue color low overlap. B: Total loadings for all input vectors with sparse k-means. Light color indicates a strong contribution to separation of the clusters and vice versa. Grey color indicates that the variable has been excluded. C: Cluster center matrix for DEPECHE analysis. Rows indicate the DEPECHE clusters, columns indicate the variables that contribute to separating at least one cluster. A light color indicates that the cluster center is located in the upper part of the distribution of values in the vector in question, and vice versa. Grey color indicates that the variable has been excluded. For DEPECHE, only 10 dimensions are shown, as the 11th did not contribute to separating any cluster. (TIF) [file pone.0203247.s001.tif]

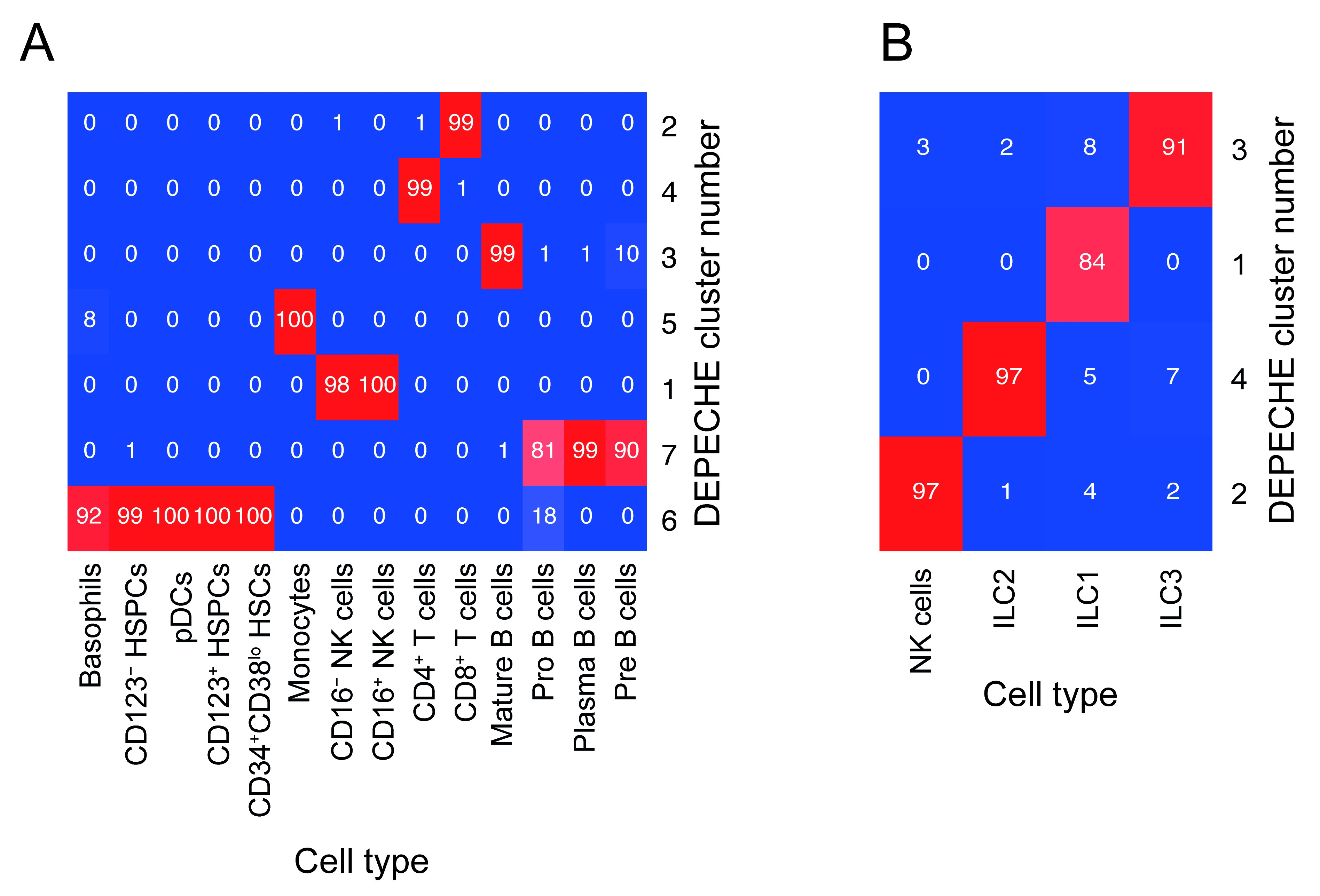

Supplement: S2 Fig — Red color indicates large overlap, blue color indicates low overlap between a gold standard-vs-DEPECHE cluster pair. Numbers in heatmaps denote percent of the golden standard cluster present in the DEPECHE cluster in question. (TIF) [file pone.0203247.s002.tif]

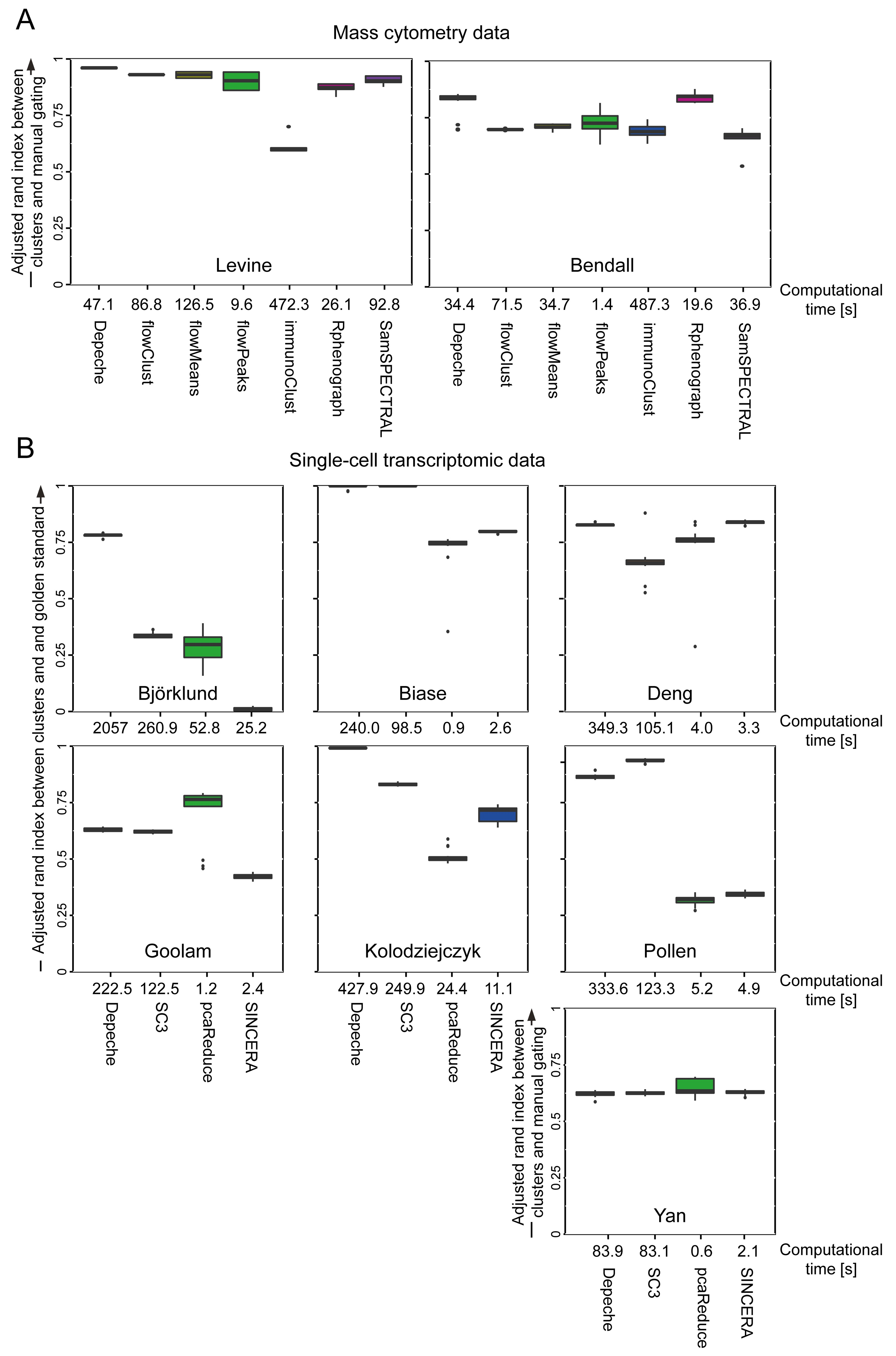

Supplement: S3 Fig — For all graphs, the x-axis shows the algorithms and the y-axis shows the Adjusted Rand Index comparing the clustering result with the golden standard clustering. Below each graph is the average computational time in seconds for the benchmarking performed on a laptop computer with 4 2.8 GHz Intel Core i7 processors. A) Subsamples with 20000 unique cells from two mass cytometry datasets published by Levine et al and Bendall et al were clustered with DEPECHE and six previously published algorithms. For each dataset and algorithm, clustering was performed on 20 unique subsamples. For flowClust, flowPeaks and SamSPECTRAL, that do not perform internal parameter tuning, a range of parameter values were evaluated and the parameter value sets generating the highest ARI values were selected for display. B) The full Björklund dataset, as well as six other datasets previously used for benchmarking by Kiselev et al were clustered 20 times with DEPECHE and three other algorithms. The Björklund dataset was normalized to reduce batch effects, with the procedure described in the original publication. These six datasets were also automatically log2-transformed within DEPECHE, and thus, log2-transformation was applied also for Sincera and pcaReduce, whereas sc3 was fed both log2- and untransformed data. The lower and upper hinges of all boxplots extend to the 25:th and 75:th percentile, whereas the line in the middle describes the median. The whiskers extend to the lowest and highest value no further than 1.5 times the distance between the 25:th and 75:th percentile. Outside of this range, the observations are considered outliers and are shown as dots. (TIF) [file pone.0203247.s003.tif]
