# Supplementary material for: Determination of essential phenotypic elements of clusters in high-dimensional entities—DEPECHE
Source: PLoS One. 2019 Mar 7;14(3):e0203247. doi: 10.1371/journal.pone.0203247 (PMC6405191; doi:10.1371/journal.pone.0203247)
Supplement: S2 File — (PDF) [file pone.0203247.s005.pdf]

Primary data sources

The dataset for Fig 1 and for S1 Fig are generated with R scripts provided in S1_file.

The Björklund dataset is available from:
https://github.com/asabjorklund/ILC_scRNAseq/blob/master/data/ensembl_rpkmvalues_ILC.txt.gz
The steps described at the following link have been performed to generate the file cb0:
https://github.com/asabjorklund/ILC_scRNAseq/blob/master/ILC_scRNA_analysis.md

All other datasets have been imported into R in their available form, with links listed below.
Levine: https://community.cytobank.org/cytobank/experiments/46102
Bendall: https://community.cytobank.org/cytobank/experiments/46259
Biase: https://scrnaseq-public-datasets.s3.amazonaws.com/scater-objects/biase.rds
Deng: https://scrnaseq-public-datasets.s3.amazonaws.com/scater-objects/deng-rpkms.rds
Goolam: https://scrnaseq-public-datasets.s3.amazonaws.com/scater-objects/goolam.rds
Kolodziejczyk: https://scrnaseq-public-datasets.s3.amazonaws.com/scater-objects/kolodziejczyk.rds
Pollen: https://scrnaseq-public-datasets.s3.amazonaws.com/scater-objects/pollen.rds
Yan: https://scrnaseq-public-datasets.s3.amazonaws.com/scater-objects/yan.rds
